# Duration and intensity of shade differentially affects mycorrhizal growth- and phosphorus uptake responses of *Medicago truncatula*

**DOI:** 10.3389/fpls.2015.00065

**Published:** 2015-02-13

**Authors:** Tereza Konvalinková, David Püschel, Martina Janoušková, Milan Gryndler, Jan Jansa

**Affiliations:** ^1^Laboratory of Fungal Biology, Institute of Microbiology, Academy of Sciences of the Czech RepublicPrague, Czech Republic; ^2^Department of Mycorrhizal Symbioses, Institute of Botany, Academy of Sciences of the Czech RepublicPrůhonice, Czech Republic

**Keywords:** arbuscular mycorrhizal symbiosis, light intensity, shading duration, mycorrhizal benefits, phosphorus acquisition, biomass production, root colonization, nitrogen fixation

## Abstract

Plant and fungal partners in arbuscular mycorrhizal symbiosis trade mineral nutrients for carbon, with the outcome of this relationship for plant growth and nutrition being highly context-dependent and changing with the availability of resources as well as with the specific requirements of the different partners. Here we studied how the model legume *Medicago truncatula*, inoculated or not with a mycorrhizal fungus *Rhizophagus irregularis*, responded to a gradient of light intensities applied over different periods of time, in terms of growth, phosphorus nutrition and the levels of root colonization by the mycorrhizal fungus. Short-term (6 d) shading, depending on its intensity, resulted in a rapid decline of phosphorus uptake to the shoots of mycorrhizal plants and simultaneous accumulation of phosphorus in the roots (most likely in the fungal tissues), as compared to the non-mycorrhizal controls. There was, however, no significant change in the levels of mycorrhizal colonization of roots due to short-term shading. Long-term (38 d) shading, depending on its intensity, provoked a multitude of plant compensatory mechanisms, which were further boosted by the mycorrhizal symbiosis. Mycorrhizal growth- and phosphorus uptake benefits, however, vanished at 10% of the full light intensity applied over a long-term. Levels of root colonization by the mycorrhizal fungus were significantly reduced by long-term shading. Our results indicate that even short periods of shade could have important consequences for the functioning of mycorrhizal symbiosis in terms of phosphorus transfer between the fungus and the plants, without any apparent changes in root colonization parameters or mycorrhizal growth response, and call for more focused research on temporal dynamics of mycorrhizal functioning under changing environmental conditions.

## INTRODUCTION

The arbuscular mycorrhizal (AM) symbiosis is one of the most abundant symbioses on Earth, and it is important for the movement of nutrients through global ecosystems. Host plants exchange carbon (C) for mineral nutrients such as phosphorus (P), nitrogen (N) and zinc provided by AM fungi (Lekberg et al., 2010; Fitter et al., 2011).

Research in the last few decades has made a great progress in understanding P acquisition pathways of plants via AM fungi (Burleigh and Harrison, 1999; Rausch et al., 2001; Javot et al., 2007). Ecophysiological experiments using P-radioisotope labeling of soil patches accessible only to the AM hyphae have demonstrated the importance of the mycorrhizal P uptake pathway for plant nutrition. This pathway had previously been massively underestimated, but it can account for nearly all P uptake by a host plant under P-limiting soil conditions (Pearson and Jakobsen, 1993a; Smith et al., 2004).

Compared to P uptake, the C side of the symbiosis is much less understood. Initial estimates suggested that approximately 4% of the plant photosynthetic production is allocated to fungal symbionts (Paul and Kucey, 1981). Further studies showed that up to 20–30% of the C budget is being consumed by the fungal partners under some circumstances (Jakobsen and Rosendahl, 1990; Drigo et al., 2010). The average values for the different combinations of partners and different environmental conditions now stand somewhat below 10% of plant photosynthesis production (Grimoldi et al., 2006; Lendenmann et al., 2011). There is a great variability in these figures, however, depending on the identity of the fungal symbionts, with some fungal partners demanding more C than others (Pearson and Jakobsen, 1993b; Lendenmann et al., 2011). It has also been demonstrated that C allocation to the fungal partner is coupled with exchange of P and/or N (Hammer et al., 2011; Kiers et al., 2011; Fellbaum et al., 2012, 2014), meaning that nutrient availability and plant requirements can change C allocation patterns.

One approach to studying C allocation dynamics is to impose experimental shading on the host plant in order to manipulate the C source strength (Fellbaum et al., 2014; Knegt et al., 2015) and to determine how this changes the C allocation to the fungal partner. Previous research on the effects of light intensity on the AM symbiosis demonstrated a reduction of symbiotic benefits under reduced light intensities and a reduced fungal colonization rate in continuously or temporarily shaded plants (Son and Smith, 1988; Reinhard et al., 1994; Tuomi et al., 2001; Heinemeyer et al., 2003; Olsson et al., 2010). Other studies, however, found no reduction in symbiotic benefits and mycorrhizal colonization (Gehring, 2003; Korhonen et al., 2004; Millar and Ballhorn, 2013).

There is little doubt that the incoming light is the only source and light intensity thus being the primary determinant of metabolic energy availability for both the plant and the associated AM fungi. However, our understanding of the quantitative changes in symbiotic functioning as depends on the light intensity is still very limited. Further, we do not know what effects will the duration of exposure to different light intensities have on the functioning of the symbiosis on the gradient of plant responses ranging from positive to negative (Johnson et al., 1997, 2015; Johnson and Graham, 2013).

Here we study mycorrhizal functioning of the model plant *Medicago truncatula* Gaertn. across a gradient of light levels. We used a model mycorrhizal fungus, *Rhizophagus irregularis* (Blaszk., Wubet, Renker and Buscot) C. Walker and A. Schüßler as the fungal partner because it has previously been shown to provide major P uptake benefits to the plant, but also to consume significant amounts of plant C (Lendenmann et al., 2011). Our aim was to assess the effects of duration and intensity of shading on the symbiotic benefits and costs of our model plants.

## MATERIALS AND METHODS

### EXPERIMENTAL DESIGN

We used barrel medic (*M. truncatula*) that was inoculated or not with *R. irregularis*. The experiment encompassed both long- (38 d) and short-term (6 d) shading with three different intensities of shade (65, 35, or 10% of the incoming light intensity), as well as a full-light control treatment. Each treatment was replicated five times, resulting in a total of 70 pots.

### BIOLOGICAL MATERIALS

The seeds of *M. truncatula* J5 were surface-sterilized in concentrated (98%) sulfuric acid for 10 min and then rapidly washed with several liters of tap water to achieve synchronous germination. The seeds were germinated on a moist filter paper at 25°C for one day.

Inoculum of *Sinorhizobium meliloti* strain 1021 was grown in TY liquid medium (Somasegaran and Hoben, 1994) on a shaker at 24°C for 3 days. The bacteria were washed with 0.5% (w:v) MgSO_4_ solution and the suspension was adjusted to the optical density of 0.7 at 600 nm (which corresponded to approximately 2 × 10^9^ cells mL^-1^).

Inoculum of the *R. irregularis*, The International Bank for the Glomeromycota (BEG) accession number 158, previously isolated from an arable field in Tänikon, Switzerland (Jansa, 2002), was produced in pot cultures with leek (*Allium porrum* L.) cv. Du Bouchet as a host plant in climate chambers (14 h photoperiod, 350 μmol photons m^-2^ s^-1^ provided by fluorescent tubes and sodium discharge lamps, 25/20°C day/night). The cultures were grown for 4 months prior to the experiment, resulting in 89% of the leek root length colonized by the AM hyphae, 59% of root length colonized by vesicles and hyphal length density in the substrate being 3.41 m g^-1^. The “mock-inoculum” for the non-mycorrhizal treatments was produced in pots with a non-mycorrhizal leek grown for four months under the same conditions as the mycorrhizal inocula. Shortly before the start of the experiment, the above-ground biomass of leek in the pots was removed, the roots were cut to pieces of about 1 cm in length and mixed back to the substrate, which was subsequently dried at room temperature and then used as the mycorrhizal or the control inoculum.

### CULTIVATION OF THE EXPERIMENTAL PLANTS

Plants were grown in 2-L pots lined with plastic mesh (opening of 1 mm) at the bottom, sterilized with 96% ethanol and filled with substrate. The substrate consisted of autoclaved quartz sand (grain size < 3 mm), autoclaved zeolite MPZ 1-2.5 (Zeopol, http://www.zeolity.cz, grain size 1–2.5 mm) and γ-irradiated soil from Litoměřice, Czech Republic (pH_H2O_ 7.88, 42% clay, 40% sand, total P 797 mg kg^-1^, water extractable P 3.3 mg kg^-1^, 2.26% C, 0.13% total N) mixed in a ratio 9:9:2 (v:v:v). The substrate was added with 2.5% (v:v) of the respective mycorrhizal or control inocula mixed thoroughly in the whole volume.

Rhizobial suspension (4 mL pot^-1^) and five germinated seeds were put in each pot about 1 cm below the surface. Surface of the substrate was kept moist with distilled water, and after 10 days, seedlings were thinned to two per pot. Plants were then watered daily with distilled water to approximately 85% water holding capacity of the substrate. Plants were grown during the summer period in the glasshouse of the Institute of Microbiology, Prague, with supplemental lighting (halogen lamps providing a photosynthetic flux density of 200 μmol m^-2^ s^-1^), extending the day length to 16 h. The plants were fertilized with a modified White mineral solution P2N3 (Gryndler et al., 1992) with reduced amount of P (5% of the original recipe), starting at two weeks after planting, and providing each pot with 50 mL of two-times concentrated solution pot^-1^ week^-1^.

Plants were exposed to shading treatments as described above. Full light corresponded to about 50% of the solar radiation outside the glasshouse. Plants were shaded by tents made of green shading fabrics used in horticulture. Pots were fully randomized in the glasshouse and they were rotated every week before application of the shade treatments, and then they were rotated within the tents.

### ^**13**^CO_**2**_ PULSE LABELING

Plants exposed to full light and 10% light intensity applied over long-term were pulse-labeled with ^13^CO_2_ 3 days before the harvest. The pots were placed in an air-tight Plexiglas chamber (0.75 m^3^) equipped with a fan to mix the inner atmosphere. Temperature, humidity, and CO_2_ concentration inside the chamber were thoroughly monitored (using Testo 435-2 datalogger equipped with the IAQ probe, Testo AG, Lenzkirch, Germany). The air in chamber was enriched with ^13^CO_2_ by adding 40% phosphoric acid onto 1 g of 99% ^13^C-enriched calcium carbonate powder (Sigma–Aldrich, Buchs, Switzerland) placed in the labeling chamber to achieve a calculated^1^ CO_2_ concentration of 574 μmol mol^-1^, while the ^13^C enrichment inside the chamber reached 57 atom% ^13^C. The labeling took 1.5 h on a sunny day, with the light intensity at plant level reaching 891 μmol photons m^-2^ s^-1^.

### HARVEST AND SAMPLING

Plants were harvested 7.5 weeks after planting. For all measurements and analyses, except the shoot architecture analysis, both plants from one pot were processed as one unit. The shoots were cut at the hypocotyl-root boundary, laid out between a PVC foil and a Plexiglas plate and photographed together with a ruler for shoot architectural analyses. Subsequently, the shoots were dried for 4 days at 65°C to determine the shoot dry weight. The roots were washed from the substrate with tap water, cut into 1-cm fragments and stirred in water. Then the roots were divided in two aliquots and the fresh weights of both were recorded. One aliquot was immersed in 10% KOH (w:v) to determine the AM fungal colonization, while the second aliquot was dried for 4 days at 65°C, weighed and the root dry weight of whole root system was calculated. The samples of substrate were stored at -80°C before being dried at 65°C for 4 days. Dried shoot, root, and substrate samples were milled to a fine powder using a ball mill (MM200, Retsch, Haan, Germany) before the elemental and isotopic analyses.

### PLANT, FUNGAL, AND SUBSTRATE ANALYSES

To determine the P concentration in plant tissues, milled samples of shoots and roots (100 mg each) were incinerated in a muﬄe furnace (550°C) for 12 h, added with 1 mL of concentrated (69%) HNO_3_ and briefly heated to 250°C on a hot plate. Then they were transferred to volumetric flasks through a filter paper and made up to 50 mL with ultrapure water. Phosphorus concentration in the extracts was then measured using the malachite green method (Ohno and Zibilske, 1991).

The N and C concentrations and isotopic composition of N and C in shoots, roots, and substrate were measured in all pots subjected to ^13^C labeling, using an elemental analyzer (Flash EA 2000) coupled with an isotope ratio mass spectrometer (Delta V Advantage, ThermoFisher Scientific, Waltham, MA, USA). The isotopic compositions were expressed in notation (‰) relative to atmospheric air standard in case of ^15^N and to Vienna Pee Dee Belemnite (VPDB) standard in case of ^13^C (Eq. 1; *R*_s_ and *R*_st_ being the isotope ratios in the sample and the standard, respectively).

(1) $$\delta \left(‰\right)=(\frac{R_{s}}{R_{st}}-1)\times 1000\,$$

Additionally, to calculate the amount of ^13^C in the samples which originated from the ^13^CO_2_ pulse (i.e., excess ^13^C), the δ values were converted to F-ratios (Eq. 2; where the stands for δ^13^C(‰) of the sample divided by 1000; 0.0111802 is the ^13^C/^12^C isotope ratio of the VPDB standard). The amounts of carbon in the roots, shoots or substrate (*C*, in moles) were calculated as in Eq. 3 (*DW* is the dry weight of the respective sample; *B* is the carbon concentration in the sample, g g^-1^; *F* is the ^13^C F-ratio in the sample). Finally, the amounts of ^13^C in the roots, the shoots or the substrate added by labeling (*excess^13^C*, in moles) were calculated (Eq. 4; *F*_s_ is ^13^C F-ratio in the sample; *F*_u_ is the average ^13^C F-ratio in the corresponding samples of five unlabeled plants).

(2) $$F=\frac{\left(\delta +1\right)\times 0.0111802}{\left(\delta +1\right)\times 0.0111802+1}\,$$

(3) $$C=\frac{DW\times B}{13\times F+12\times \left(1-F\right)}\,$$

(4) $${excess}^{13}C=\left(F_{s}-F_{u}\right)\times C\,\;\;\;\;\;\;\;\;\;\;\;\;\;\;\;\;\;\;\;\;\;\;\;\;\;\;\;\;\;\;\;\;\;\;\;\left(4\right)$$

Root samples for staining were first macerated in 10% KOH (30 min at 90°C), washed with tap water, neutralized in 1% HCl (1 h at room temperature followed by 15 min at 90°C) and stained with a mixture of Trypan and Methylene Blue (each 0.05% in lactic acid–glycerol–water, 1:1:1 v:v:v). The AM fungal colonization was assessed microscopically using the method of McGonigle et al. (1990).

Shoot architecture was assessed on each individual plant with image analyzing software (NIS-Elements AR, Nikon Instruments, Melville, NY, USA). The length of the shoot main axis and the number of branches were averaged for each pot using the values measured on the two individual plants. The mean surface of leaflets was calculated as an average of 18 measured leaflets per pot.

### STATISTICAL ANALYSIS

The data were analyzed with Statgraphics Plus for Windows version 3.1. The shoot and root weights, P and N contents, P concentrations, and the leaflet surface values were common log-transformed, whereas the root to shoot biomass ratios and excess^13^C values were square root-transformed. Fractional values of the mycorrhizal colonization of roots were arcsin (square root)-transformed before the analyses. Mycorrhizal responses were calculated as in Thonar et al. (2011). Data from long- and short-term shading treatments were analyzed separately.

Simple regression analyses using a linear model were performed to test the influence of light intensity on *Rhizophagus-*colonization of roots and on the mycorrhizal responses. General linear models (GLM) were employed to test the significances of the effects of inoculation treatment and light intensity on the plant dry weights, P contents and concentrations and plant architectural parameters, while two-way ANOVAs with inoculation treatment and light intensity as factors were carried out for the carbon and nitrogen data, because they were only collected for the full light and for the most intensive long-term shading treatments. The significance of individual factors and their interactions were judged by F-ratios. The effect was considered significant if *p* < 0.05.

Additionally, *t*-tests were performed to test the effect of mycorrhizal inoculation separately for the full light and for the 10% light intensity applied over long-term. Mann–Whitney test replaced the *t*-test comparisons if significant heteroscedasticity in the data was detected.

## RESULTS

### PLANT GROWTH

**FIGURE 1:**
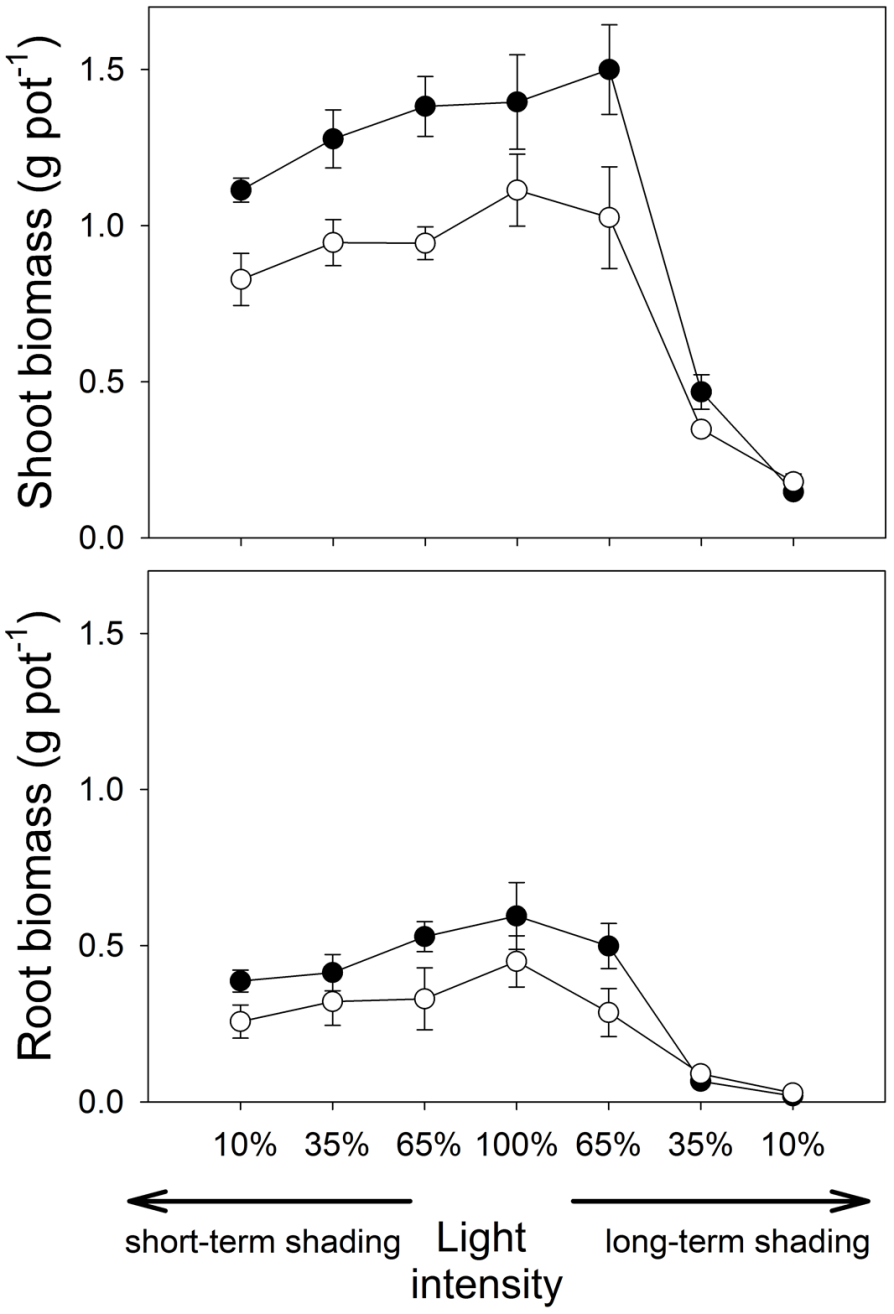
**Shoot and root dry biomass of *Medicago truncatula* plants inoculated or not with *Rhizophagus irregularis* (black and white symbols, respectively), and subjected to different light regimes**. Mean values ± SD (*n* = 5) are shown.

At full light, both shoot and root dry weights (**Figure 1**) were higher for the mycorrhizal than non-mycorrhizal plants (*t*_8_ = 3.43, *p* = 0.009 and *t*_8_ = 2.32, *p* = 0.048, respectively).

Under short-time shading, both shoot and root weights decreased significantly with the decrease of light intensity (*F*_1,36_ = 44.2, *p* < 0.001, and *F*_1,36_ = 39.2, *p* < 0.001, respectively). However, the differences between the inoculation treatments did not change significantly with light intensity as revealed by a non-significant interaction between the inoculation and the light factors (Supplementary Table S1).

Under long-term shading, the response of shoot weight to light intensity was not trivial – there was only a small difference between full light and 65% light intensity (**Figure 1**), while plant biomass production was strongly reduced under further shading levels. Therefore, there was a strong effect of light treatment on the shoot weight (*F*_1,36_ = 186, *p* < 0.001), but the effect of inoculation treatment and the interaction of the main factors were both not significant. In contrast to the full light treatment, non-mycorrhizal plants had higher shoot biomass than mycorrhizal plants at 10% light intensity applied over long time (*t*_8_ = 2.46, *p* = 0.039, **Figure 1**; **Table 1**). Root biomass also decreased significantly with the decrease of light intensity in the long-term shading (*F*_1,36_ = 296, *p* < 0.001). The differences in root biomass between the inoculation treatments were reversed at low light levels as compared to the full light, as indicated by a significant interaction between the inoculation and light factors (*F*_1,36_ = 5.58, *p* = 0.024). Indeed, at 10% light intensity applied over a long term, higher root weight was recorded for the non-mycorrhizal plants as compared to the mycorrhizal treatment (*W* = 0.00, *p* = 0.012).

**Table 1:** *Medicago truncatula* responses to mycorrhizal inoculation under the different light regimes.

Shading duration	Long-term			None	Short-term		
Light intensity	10%	35%	65%	100%	65%	35%	10%
Dry shoot biomass	-18	35	46	25	46	35	35
Dry root biomass	-35	-30	74	32	60	29	51
Shoot P content	-1	106	130	145	117	102	52
Root P content	-13	39	347	197	357	207	289
Shoot P concentration	21	53	56	96	48	51	12
Root P concentration	35	95	155	125	15	145	155

*Mean values (*n* = 5) are shown of the mycorrhizal responses given as a mean percent change to the respective non-mycorrhizal control treatment.*

### PHOSPHORUS UPTAKE

**FIGURE 2:**
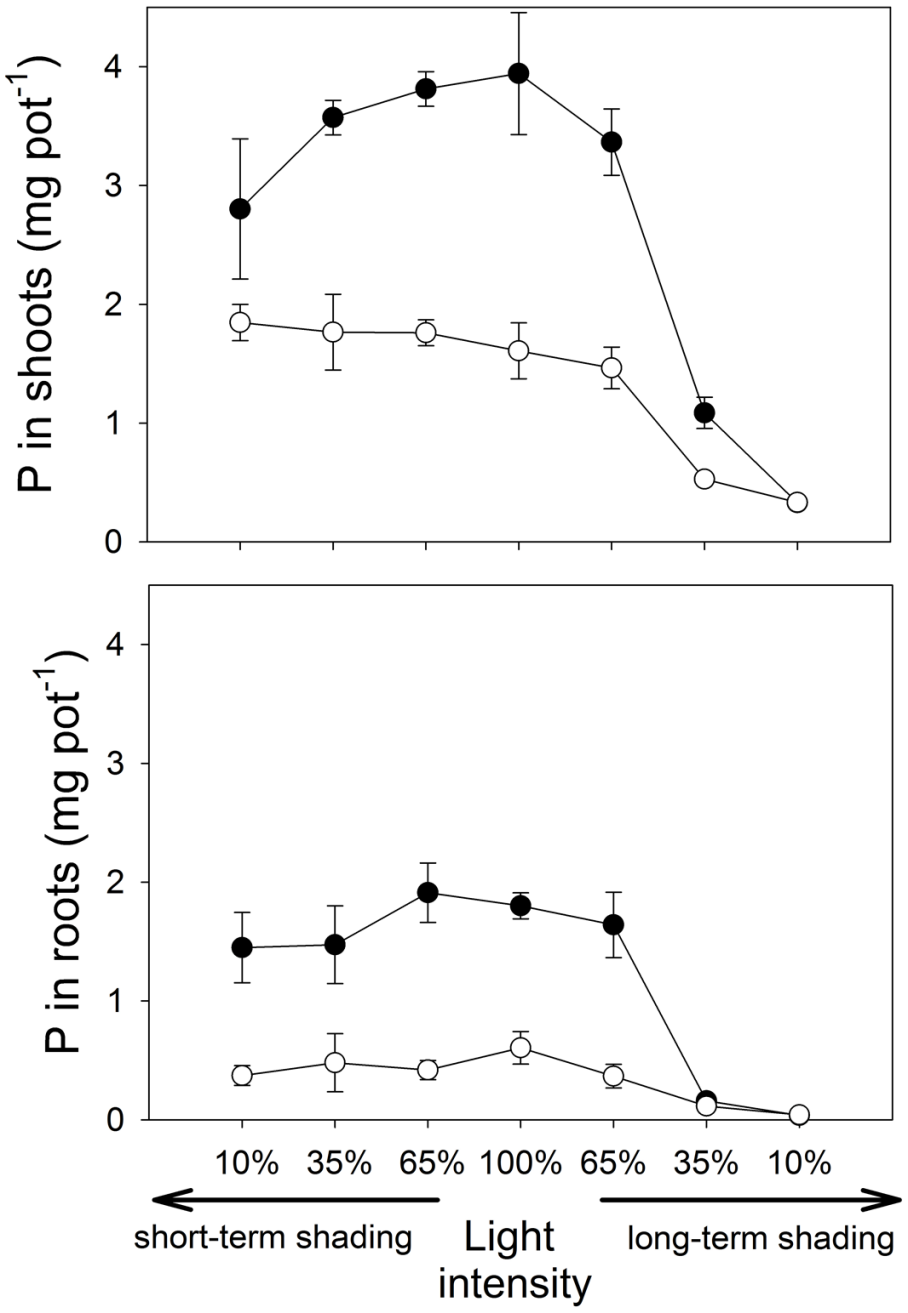
**The amount of phosphorus (P) contained in the shoots and roots of *M. truncatula* plants inoculated or not with *R. irregularis* (black and white symbols, respectively), and subjected to different light regimes.** Mean values ± SD (*n* = 5) are shown.

**FIGURE 3:**
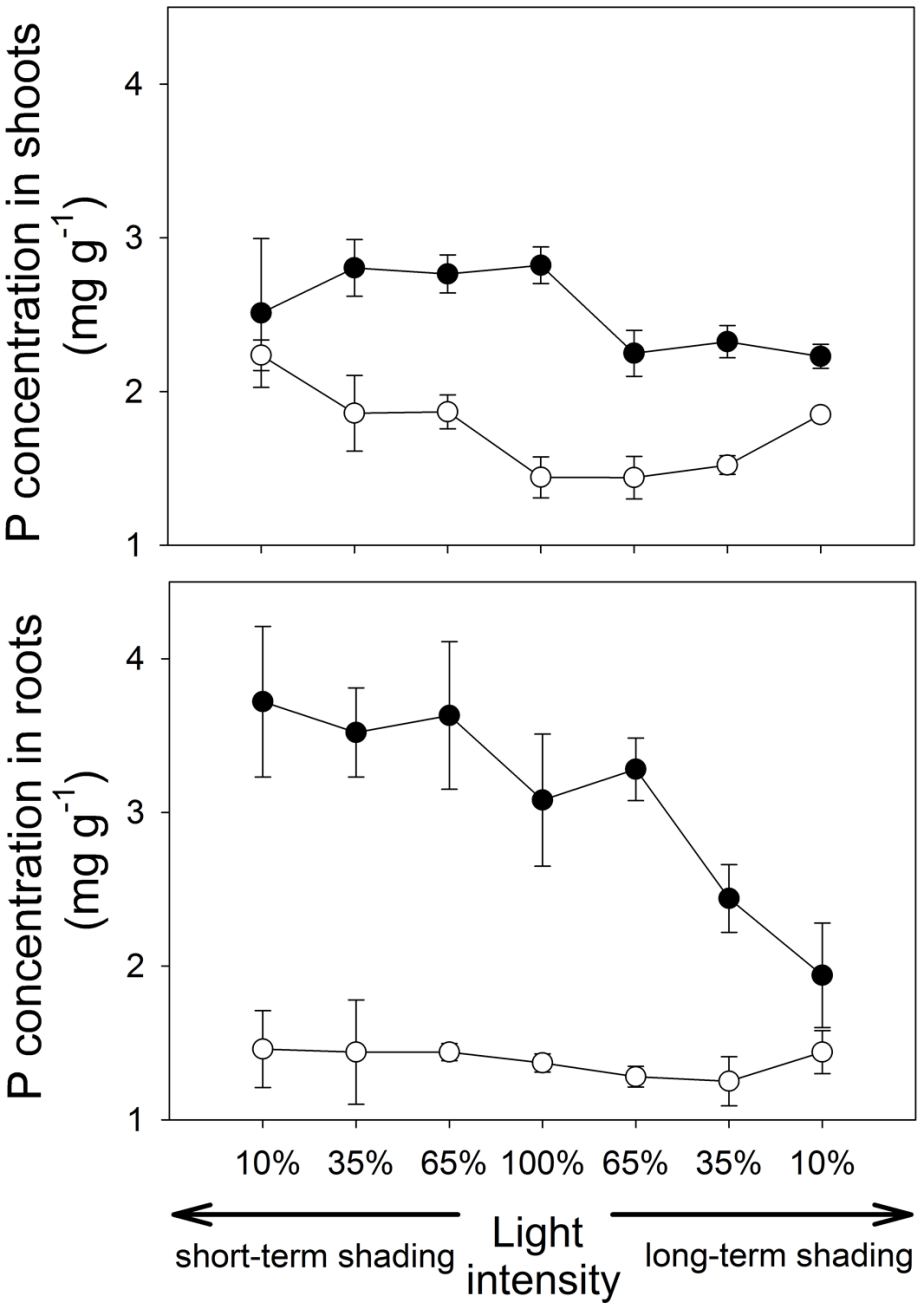
**Phosphorus (P) concentrations in shoots and roots of the *M. truncatula* plants inoculated or not with *R. irregularis* (black and white symbols, respectively), and subjected to different light regimes.** Mean values ± SD (*n* = 5) are shown.

At full light, P contents in shoots and roots (**Figure 2**) were significantly higher in mycorrhizal than the non-mycorrhizal plants (*t*_8_ = 10.5, *p* < 0.001 and *W* = 20.0, *p* = 0.020, respectively). Phosphorus concentrations in both shoots and roots (**Figure 3**) were also higher in mycorrhizal as compared to the non-mycorrhizal plants (*t*_8_ = 15.4, *p* < 0.001 and *t*_7_ = 10.9, *p* < 0.001, respectively).

Under short-term shading, the responses of shoot P content to light intensity strongly depended on the inoculation treatment, as revealed by a significant interaction between the inoculation and light factors (*F*_1,36_ = 15.8, *p* < 0.001). Whereas the shoot P content of mycorrhizal plants decreased with decreasing light intensity, the P content of shoots of the non-mycorrhizal plants remained unaffected by the shading (**Figure 2**), resulting in a significant decrease of the mycorrhizal shoot P content response with decreasing light intensity (*F*_1,18_=35.7, *p*<0.001, **Table 1**). The response of the root P content to the short-time shading was independent of the inoculation treatment, as revealed by a non-significant interaction between the inoculation and light factors and by a non-significant correlation of the mycorrhizal root P content response with light intensity (Supplementary Table S1; **Table 1**). Due to slowing biomass production of but continuing P uptake to the short-term shaded non-mycorrhizal plants, the P concentration in the shoots of non-mycorrhizal plants increased with increasing intensity of short-term shading, whereas the P concentration in the mycorrhizal plants stagnated or tended to decrease (**Figure 3**). These effects were significant as revealed by a significant interaction of the light and inoculation factors (*F*_1,36_ = 30.9, *p* < 0.001) and by a significant correlation of the mycorrhizal P concentration response with light intensity. Such a difference in response to shading between the inoculation treatments was not replicated in the root P concentrations (**Figure 3**), as revealed by a non-significant interaction between the light and inoculation treatments.

Under long-term shading, both shoot and root P contents were significantly reduced (*F*_1,36_ = 234, *p* < 0.001 and *F*_1,35_ = 251, *p* < 0.001, respectively). The decrease was most pronounced between 65 and 35% of the full light intensity and it was more rapid in mycorrhizal than in the non-mycorrhizal plants, as revealed by a significant interaction between the inoculation and light factors (*F*_1,36_ = 8.76, *p* = 0.005 and *F*_1,35_ = 9.88, *p* = 0.003, respectively) and by significant correlations between mycorrhizal P content responses in shoots and roots and the light intensity (*F*_1,18_ = 37.1, *p* < 0.001 and *F*_1,18_ = 16.5, *p* < 0.001, respectively). No significant differences were found between the inoculation treatments in the P content of either shoots or roots at 10% light intensity applied over a long-term. The P concentrations in both shoots and roots decreased significantly more along the shading gradient in the mycorrhizal as compared to the non-mycorrhizal plants, as documented by a significant interaction between the light and inoculation factors for both parameters (*F*_1,36_ = 41.1, *p* < 0.001, and *F*_1,35_ = 19.1, *p* < 0.001, respectively) and by a significant correlation of the mycorrhizal P concentration response for both shoots and roots with light intensity (*F*_1,18_ = 117, *p* < 0.001 and *F*_1,18_ = 19.0, *p* < 0.001, respectively).

### NITROGEN UPTAKE

Shoot N content was not affected by the inoculation (Supplementary Table S1), but only by long-term shading (*F*_1,16_ = 773, *p* < 0.001). Nevertheless, δ^15^N values (Supplementary Figure S1), indicative of the origin of N in shoot biomass, differed between the inoculation treatments at full light (*W* = 0.00, *p* = 0.012). Mycorrhizal plants had lower δ^15^N value, indicating a higher proportion of symbiotically fixed nitrogen than in the non-mycorrhizal plants. This pattern was reversed in the plants exposed to 10% light intensity over a long term, as revealed by a significant interaction between the inoculation and light factors for the δ^15^N in the shoots (*F*_1,16_ = 51.2, *p* < 0.001). At 10% light intensity, mycorrhizal plants showed higher δ^15^N value than the non-mycorrhizal controls (*t*_8_ = 14.8, *p* < 0.001), indicating a lower proportion of symbiotically fixed N than in the non-mycorrhizal plants.

### PLANT ARCHITECTURE

**FIGURE 4:**
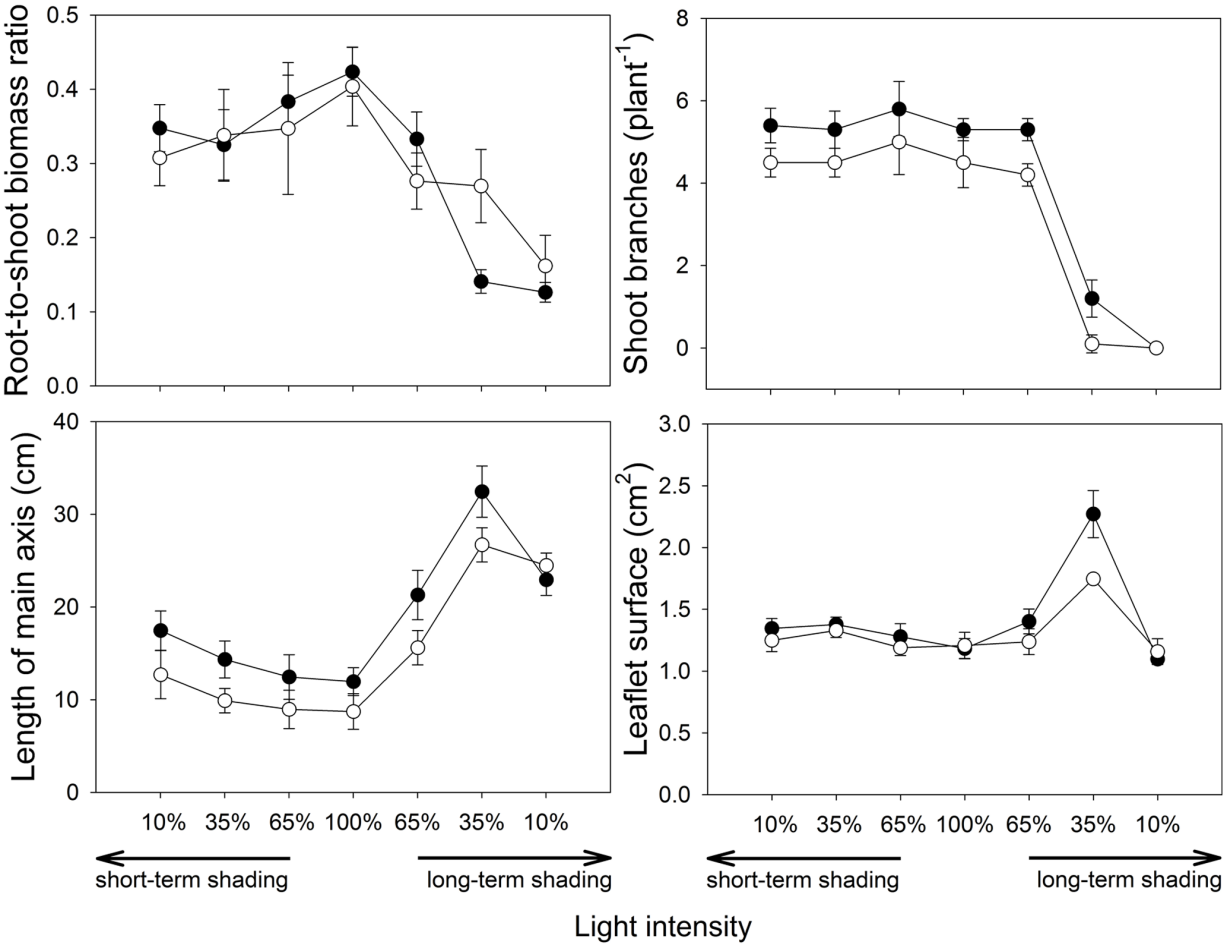
**Architecture of *M. truncatula* plants inoculated or not with *R. irregularis* (black and white symbols, respectively), and subjected to different light regimes.** Mean values ± SD (*n* = 5) are shown.

The root-to-shoot biomass ratio (**Figure 4**) did not differ between the inoculation treatments at full light and decreased with decreasing light intensity under both short- and long-term shading regimes (*F*_1,36_ = 16.7, *p* < 0.001 and *F*_1,36_ = 183, *p* < 0.001, respectively). The response to long-term shading was strongly affected by the inoculation treatment, as revealed by a significant interaction between the inoculation and light factors (*F*_1,36_ = 8.43, *p* = 0.006). There was a sharp drop of root-to-shoot biomass ratio between 65 and 35% light intensities in mycorrhizal plants, while the decrease was more gradual in the non-mycorrhizal plants, which consequently had a considerably higher root-to-shoot ratio than the former at 35% light intensity applied over a long-term (*t*_8_ = 5.98, *p* < 0.001). However, no significant difference among inoculation treatments was detected at 10% light intensity applied over a long-term.

Numbers of shoot branches (**Figure 4**) was higher in mycorrhizal than non-mycorrhizal plants at full light (*F*_2,12_ = 10.9, *p* = 0.002) and this parameter was unaffected by short-term shading. Under long-term shading, the number of branches decreased with decreasing light intensity (*F*_1,36_ = 177, *p* < 0.001), and this decrease was most pronounced between 65 and 35% light intensities. No plants had side branches at 10% light intensity applied over a long-term.

The length of the main axis (**Figure 4**) depended strongly on the inoculation treatment under full light (*t*_8_ = 2.94, *p* = 0.019), with higher values observed in the mycorrhizal plants. The length of axis increased under both short- and long-term shading (*F*_1,36_ = 26.5, *p* < 0.001 and *F*_1,36_ = 66.6, *p* < 0.001, respectively). Furthermore, under long-term shading, a shortening of the longest branch was apparent between 35 and 10% light intensities, more pronouncedly in the mycorrhizal treatment, thus no difference among inoculation treatments was eventually observed at 10% light intensity.

The mean leaflet surface (**Figure 4**) did not differ among the inoculation treatments at full light. It was slightly but significantly increased by the short-term shading (*F*_1,36_ = 27.7, *p* = 0.001) and there was also a slight increase of the leaflet surface due to the mycorrhizal inoculation in shortly shaded plants (*F*_1,36_ = 4.29, *p* = 0.046). The response of leaflet surface to long-term shading was not monotonous, thus no significant effects were found there using the GLM analysis. Nevertheless, further GLM analysis with 100, 65 and 35% light intensities only applied over a long-term showed that leaflet surfaces strongly increased with decreasing light intensity (*F*_1,26_ = 97.1, *p* < 0.001) and this increase was intensified by mycorrhizal inoculation, as revealed by a significant interaction between the inoculation and the light factors (*F*_1,26_ = 7.45, *p* = 0.011). Leaflet surface at 10% light intensity was then approximately the same as at full light, and it did not differ between the inoculation treatments.

### MYCORRHIZAL COLONIZATION

**FIGURE 5:**
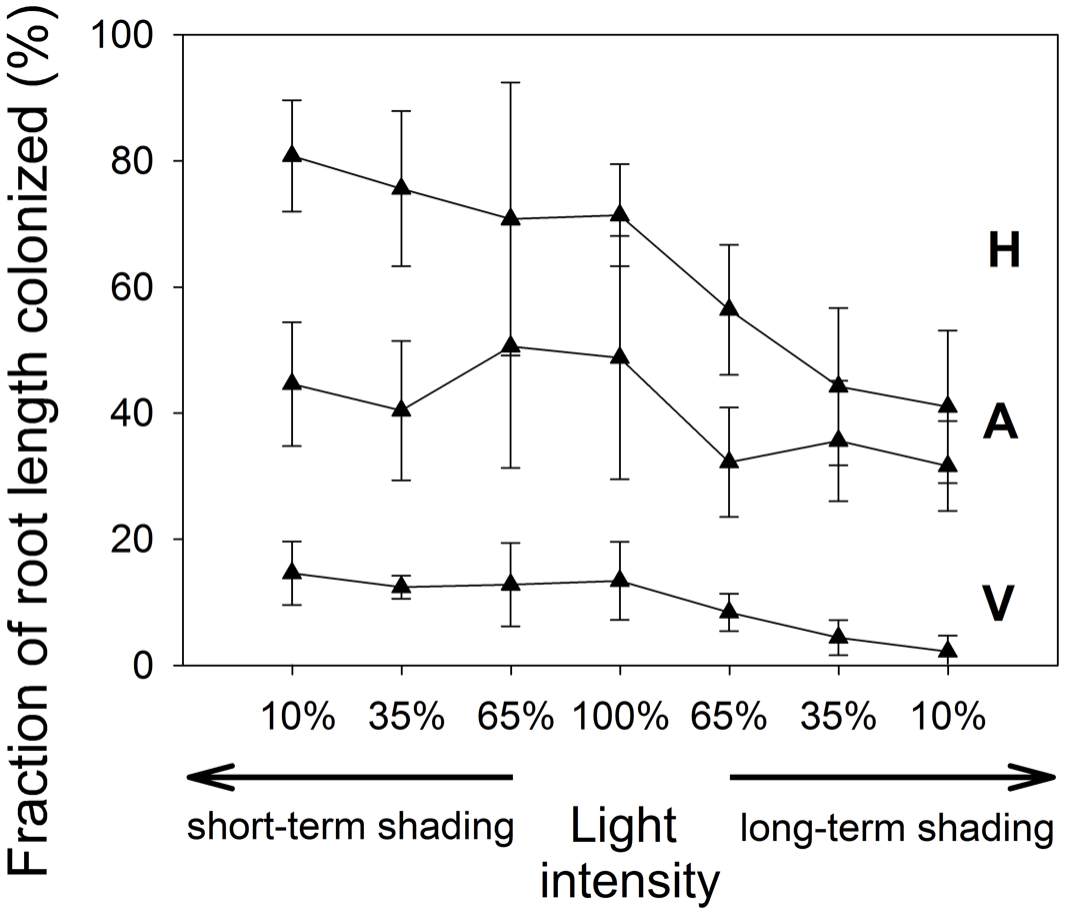
**The extent of root length colonized by *R. irregularis* in the inoculated *M. truncatula* plants subjected to different light regimes.** Mean values ± SD (*n* = 5) are shown. H, colonization by mycorrhizal hyphae; A, colonization by arbuscules; V, colonization by vesicles.

The extent of root colonization by the mycorrhizal fungus in the *Rhizophagus*-inoculated treatment (**Figure 5**) varied between 34 and 96% of the root length colonized by AM fungal hyphae. Extent of root colonization by hyphae, vesicles, and arbuscules did not change significantly under short-term shading, but the first two decreased significantly under long-term shading (*F*_1,18_ = 25.1, *p* < 0.001 and *F*_1,18_ = 29.2, *p* < 0.001, respectively), whereas the decrease of the root occupancy by arbuscules was only marginally significant (*p* = 0.068). There were no AM fungal structures detected in the roots of non-mycorrhizal plants (data not shown).

### CARBON ALLOCATION

Both shoot and root ^13^C excess values (Supplementary Figure S2) showed a similar pattern as the shoot and root biomass data, although the differences between the two inoculation treatments were not statistically significant at any of the two light intensities applied over a long time. Both parameters were significantly higher at full light than at 10% light intensity applied over long-term (*F*_1,16_ = 510, *p* < 0.001 and *F*_1,16_ = 597, *p* < 0.001, respectively). Significant interaction between the light intensity and inoculation for the root ^13^C excess (*F*_1,16_ = 5.76, *p* = 0.029) indicates proportionally greater allocation of the ^13^C from the labeling pulse to the roots of unshaded mycorrhizal plants, while the reverse was observed in the shaded plants (Supplementary Figure S2). Simultaneously to root-to-shoot biomass ratios, the root-to-shoot ratios of ^13^C excess decreased with decreasing light intensity applied over a long-term (*F*_1,16_ = 338, *p* < 0.001) and this decrease was more pronounced in mycorrhizal than in the non-mycorrhizal plants, as revealed by a significant interaction of the inoculation and light treatments (*F*_1,16_ = 5.75, *p* = 0.029). The excess ^13^C in the substrate did not depend on any of the tested factors (data not shown).

## DISCUSSION

This research highlighted several important features in the responses of plant-AM fungal symbiosis to shading with different intensities and durations. These results improve our understanding of phosphorus and carbon economy of mycorrhizal legumes along a gradient of light availabilities, and generate testable hypotheses for further studies. Here is the summary of the most important observations:

(1) In mycorrhizal plants subjected to a sudden light deprivation, P acquisition to the shoots rapidly declined, and the decline was proportional to the intensity of shade. Such a decline was not observed in the non-mycorrhizal plants. In contrast, there was no indication of a decline of the P flow to the roots of mycorrhizal plants via AM hyphal networks as compared with the non-mycorrhizal plants, with the gradually raising P concentration in the roots of the mycorrhizal plants indicating P accumulation in the intraradical fungal tissues.
(2) Long-term shading, particularly if more than 50% of the light was cut off, resulted in a significant decline of plant biomass production and massive rearrangements of plant architecture, indicating a strong C limitation. These morphological adaptations were particularly boosted by the presence of the AM fungus, but only to a certain level of light deprivation. At the most extreme shading intensity applied over a long-term, positive effects of the mycorrhizal symbiosis on the plant growth, nutrition, as well as architectural adaptations completely vanished. Root colonization levels by *R. irregularis* also declined with increasing intensity of shade applied over a long-term.
(3) Isotopic composition of shoot N in the plants inoculated with *R. irregularis* indicated an important redirection of C fluxes toward the root symbionts when the host plant was intensively shaded over a long period of time. In comparison with the non-mycorrhizal plants, *Rhizophagus*-inoculated plants seemed to have benefited from rhizobial symbiosis more at full light than when shaded. The N-isotopic data thus provide indirect evidence that the C flux from the shoots has been preferentially diverted to the mycosymbiont at the expense of the rhizobia when the plant was shaded.

### PHOSPHORUS FLUXES

Our model plant (*M. truncatula*) is known to rely strongly on the mycorrhizal P uptake pathway for its P acquisition (Smith et al., 2004). In concert with this fact, concentrations of P in the mycorrhizal plants, especially in the roots, highly exceeded those in non-mycorrhizal plants (**Figure 3**). The discrepancy of P responses in roots and shoots in plants exposed to a short-term shading provides strong evidence that sudden light deprivation will markedly slow down the flow of P from the fungus to the plant: As the shade intensifies, the concentration of P in the roots does not decrease or even tends to increase, strengthening the mycorrhizal P concentration response for the roots. On the other hand, a significant decrease of the mycorrhizal P concentration response was observed in the shoots with increasing shade intensity (**Table 1**). It is well known that once in the plant tissues, P is rapidly redistributed within the entire plant body, effectively counteracting local P accumulation (Jeschke et al., 1997; Huve et al., 2007). Thus, the selective accumulation of P in the roots can only be explained by P contained in the AM fungal tissues, presumably as long-chain polyphosphates (Viereck et al., 2004). As the P uptake in our mycorrhizal plants was to some extent in the luxurious range, i.e., P acquisition was not the primary and immediate limitation of plant growth, rapid reduction of P transfer from the fungus to the plant did not immediately affect the growth of mycorrhizal vs. control plants. However, further shading may have had negative impact on plant growth as the P concentrations in the shoots were almost equalized between the mycorrhizal and non-mycorrhizal plants after just one week of shading.

Shading applied over a long-term not only dramatically reduced plant growth, but it also markedly decreased P concentration in the shoots and roots of the mycorrhizal plants, whereas the decrease in P concentration with increasing shade was virtually not existent in the non-mycorrhizal plants (**Figure 3**). This can be explained by reduced efficiency of the mycorrhizal P uptake pathway upon long-term C-limitation. Mycorrhizal plants thus may suffer, under intensive and long-term shading, not only from energy shortage (which is likely to be more severe in mycorrhizal as compared to the non-mycorrhizal plants) but also from reduced P uptake rates, since the plant usually down-regulate the direct P uptake pathway in response to colonization with AM fungi (Burleigh and Bechmann, 2002). However, since the P uptake per unit of biomass remained higher in the mycorrhizal than in the non-mycorrhizal plants at all levels of shading, there are still net P benefits of the mycorrhizal symbiosis detectable at all shading levels applied in our study. This means that the plant C investments into the fungus were always rewarded by an enhanced P uptake. To completely shut down the operation of mycorrhizal exchange of P for C, one may thus need to apply even greater intensities of shade than reported here.

To gain deeper and more direct insights into the P fluxes between soil, mycorrhizal fungi and the plants, radioisotope labeling would offer more specific tool to separate and quantify the P flows via the direct (root) and the mycorrhizal uptake pathways (Frossard et al., 2011). Although we did not use this direct tracing in our current study, we believe that the magnitude of the effects presented here and the clear and consistent influence of the light intensity gradient on the mycorrhizal P uptake and P concentration responses justify the above conclusions. We also hope that our results would stimulate further studies aiming at understanding the responses of mycorrhizal plants to energy deprivation at different temporal scales and advocate using both P and C isotope labeling to disentangle the rearrangement of the respective fluxes between the fungi and the plant in response to sudden environmental changes.

### GROWTH, CARBON, AND NITROGEN FIXATION

Shading applied over a long-term greatly impacted the growth and architecture of the plants, with strong compensatory mechanisms (plant elongation and increasing leaf surface) being activated upon intermediate shading levels and further stimulated by the mycorrhizal symbiosis. However, below a certain threshold (35% light intensity), the carbon costs of the symbiosis outweighed any biomass or plant architectural benefits and caused a significant growth depression of the mycorrhizal plants. The extreme shading levels tested here (10% light intensity) were still ecosystem relevant as they corresponded to the light received by understory plants under a closed (temperate to boreal) forest canopy as compared to an open landscape (Martens et al., 2000), or to extended periods of cloudy weather, or events of atmospheric smoke or volcanic ash accumulation, as compared to open skies (McCormick et al., 1995; Cirino et al., 2014).

In response to C limitation imposed by long-term shading, the plants obviously down-regulated the extent of root colonization by the AM fungus, most probably due to high C costs of the symbiosis (Son et al., 1988; Reinhard et al., 1994; Grman, 2012). Interestingly though, the colonization of roots was by far not eliminated, as also shown in other recent reports (Fellbaum et al., 2014; Knegt et al., 2015). Why and how plants still maintain relatively high colonization levels of their roots even when they are strongly C-limited and not connected to neighboring plants via common mycorrhizal networks (Lekberg et al., 2010; Walder et al., 2012; Fellbaum et al., 2014; Knegt et al., 2015) thus remains to be explained – either this is due to only a limited capacity of the host to repel the AM fungi from its roots or the fungus is retained as an insurance for a sudden change of environmental conditions.

Partly, the lack of a mycorrhizal growth depression at intermediate shading levels could possibly be attributed to the intensification of plant photosynthesis rates due to AM symbiosis establishment, a phenomenon described in a handful of experiments carried mainly under artificially lit chambers with only a fraction of a full sunlight intensity (Wright et al., 1998; Kaschuk et al., 2009, 2010). This is certainly a topic worth further investigations, advocating different light levels and temporal patterns of shading to be carefully incorporated into the experimental designs rather than scrutinizing interactions between mycorrhiza and photosynthesis up-regulation at a single light intensity as done previously. This is because some effects may only appear at certain light intensities or at a certain light regime and not others, as we also demonstrate here.

Another plant adaptation for higher C demand of the mycosymbiont under light-limiting conditions may be an increase of photosynthetically active area. Root-to-shoot biomass ratio of *Medicago* declined with decreasing intensity of light while mean leaflets area increased (except at the lowest light intensity applied over a long-time) and these morphological adaptations were more pronounced in the mycorrhizal plants at the 35% light intensity as compared to their non-mycorrhizal counterparts (**Figure 4**). This might be a reason why the plant still benefited from the symbiosis (in terms of biomass production and improved P acquisition) at this light-level despite the high costs of the symbiosis. To our knowledge, our observation is the first documented evidence of a clear morphological adaptation to light deprivation boosted by the symbiotic fungi.

The relative increase of costs of the AM symbiosis for the host plant upon decreasing light intensity could have also been buffered (partly or fully) by a reduction of symbiotic nitrogen fixation, which may be more C costly than the AM symbiosis (Paul and Kucey, 1981; Voisin et al., 2013). Our results provide indirect support for such a scenario, using the ^15^N natural abundance approach (George et al., 1993). Partitioning of C between the N- and P-supplying symbionts is likely to be regulated by the demand for these macronutrients (Mortimer et al., 2009; Wang et al., 2011). Thus, under conditions of suboptimal P-supply – here induced by lower efficiency of the mycorrhizal P-uptake pathway due to imbalance of symbiotic reward – the C is preferentially allocated to the fungus, resulting in increasing the share of N originating from the soil and/or N fertilizer rather than the N derived from the symbiotic N fixation (Voisin et al., 2013). Multitude of the different fluxes and dependencies make the situation rather complex (Supplementary Figure S3) and further studies on C partitioning between different root symbionts and its dynamics under changing environmental conditions are certainly warranted.

In this study, we detected surprisingly weak change in allocation of recent (^13^C labeled) photosynthates belowground (Root-to-shoot excess ^13^C) due to the mycorrhizal symbiosis along the light intensity gradient. This may be, on the one hand, due to the rather small share of C allocated to the fungus (Paul and Kucey, 1981; but Lendenmann et al., 2011) or, more likely, due to some important component missing from the C budget. Although we measured the ^13^C excess in the cultivation substrate next to our plants, it did not show any dependence on the mycorrhizal status of the plants. Given the short duration of labeling, it is unlikely that measuring C specifically allocated to the extraradical mycelium by means of signature fatty acids (Olsson et al., 2010) will bring much different view. However, we missed a potentially important fraction of C in this experiment, which is the one rapidly respired from the rhizosphere within a few days after labeling (Grimoldi et al., 2006; Lendenmann et al., 2011). Therefore, it seems that the delay of three days between labeling and harvest may be not the most appropriate timing to measure the mycorrhizal C drain belowground without accounting for belowground respiration. Alternative explanation would be that the amount of C allocated to the fungus was relatively small as compared to the C stored in plants. As there is still only a limited knowledge on the absolute numbers of C allocation to the fungus in mycorrhizal symbiosis, this scenario cannot be ruled out yet, but certainly deserves a dedicated attention in the future.

### ECOSYSTEM RELEVANCE OF THE RESULTS

The most surprising observation of this study was that just a few days of intense but still realistic levels of shading obviously interfered with the exchange of P for C between the plant and the AM fungus, although the P continued to be taken up from the soil by the AM fungus and transported toward the roots via the hyphae. This effectively confirms the observations made previously in a simplified *in vitro* systems (Lekberg et al., 2010; Hammer et al., 2011), but with a more realistic whole-plant model. It also indicates that the fungus has sufficient energy reserves to feed the P uptake and transport machinery over a period of several days independently from the C input from the host plant, because in our system there was no other (better quality) host plant available to feed the mycorrhizal network as in the other recent studies (Fellbaum et al., 2014; Knegt et al., 2015).

What do these results mean in a real-world? Possibly, a few cloudy days in a row or temporary shading due to industrial smoke, smog, or volcanic ash may significantly slow-down the exchange of goods at the symbiotic interface, without swiftly affecting the root colonization levels and with possibly both partners remaining prepared to restore the functioning of the interaction. For the conditions of long-term shading, frequently encountered in many ecological situations, we show that mycorrhizal fungi may be able to maintain significant levels of root colonization and that the plants are likely to possess mechanisms to compensate for the C demands of the mycosymbionts, at least up to a certain degree. This appears, again, as an important pre-requisite for an adequate reaction to changing environmental conditions: The mycorrhizal plant is thus not particularly disadvantaged in comparison with a potential non-mycorrhizal competitor and can quickly start profiting from already established mycorrhizal symbiosis – provided the conditions, especially the energy inputs, are favorable for expression of the symbiotic benefits.

Given the above thoughts and lack of specific knowledge, our results call for more detailed studies on the dynamics of mycorrhizal functioning, especially the carbon fluxing from plants to its microbial symbionts, in rapidly changing (micro-) climatic conditions – and this is what is very rarely done in glasshouse experiments (Olsson et al., 2010). Further we showed that the responses of plants to mycorrhizal symbiosis across a light intensity gradient are often not trivial, advocating specific efforts to be dedicated to manipulation of light intensity levels and shading durations in future studies on mycorrhizal ecophysiology. This appears important in order to dissect the mechanisms and quantify the volumes of carbon-for-phosphorus exchanges between the partners in mycorrhizal symbiosis as well as their control mechanisms.

## Conflict of Interest Statement

The authors declare that the research was conducted in the absence of any commercial or financial relationships that could be construed as a potential conflict of interest.

## Footnotes

1 Infrared CO_2_ sensor is differentially sensitive to ^13^CO_2_ and ^12^CO_2_. Therefore, total CO_2_ concentration of highly ^13^C enriched atmosphere cannot be measured directly, but we rather summed the CO_2_ concentration measured just before the labeling with calculated surplus of ^13^CO_2_ released from the ^13^C-carbonate.
