# Supplementary material for: Duration and intensity of shade differentially affects mycorrhizal growth- and phosphorus uptake responses of Medicago truncatula
Source: Front Plant Sci. 2015 Feb 13;6:65. doi: 10.3389/fpls.2015.00065 (PMC4327418; doi:10.3389/fpls.2015.00065)
Supplement: Supplementary file 1 [file Data_Sheet_1.DOCX]

***Supplementary Material***

**Duration and intensity of shade differentially affects mycorrhizal growth- and phosphorus uptake responses of *Medicago truncatula***

**Tereza Konvalinková^1^*, David Püschel^1,2^, Martina Janoušková^1,2^, Milan Gryndler^1^, Jan Jansa^1^**

^1^Laboratory of Fungal Biology, Institute of Microbiology, Academy of Sciences of the Czech Republic, Prague, Czech Republic

^2^Department of Mycorrhizal Symbioses, Institute of Botany, Academy of Sciences of the Czech Republic, Průhonice, Czech Republic

*** Correspondence:** Tereza Konvalinková, Laboratory of Fungal Biology, Institute of Microbiology, Academy of Sciences of the Czech Republic, Vídeňská 1083, Prague, 142 20, Czech Republic.
konvalinkova@biomed.cas.cz

1. **Supplementary Figures and Tables**

## Supplementary Tables

**Supplementary Table 1. Summary of the results of GLM analyses showing the effects of inoculation, shading and their interaction on the different plant and mycorrhizal fungal parameters.** * (p<0.05), * * (p<0.01), * * * (p<0.001), n.s. – not significant.

| Parameter | Factor | Long-term shading | Short-term shading |
| --- | --- | --- | --- |
|  |  |  |  |
| Shoot dry weight | light | F_1,36_=185 *** | F_1,36_=44.2 *** |
|  | inoculation | n.s. | F_1,36_=43.7 *** |
|  | interaction | n.s. | n.s. |
|  |  |  |  |
| Root dry weight | light | F_1,36_=296 *** | F_1,36_=39.2 *** |
|  | inoculation | n.s. | F_1,36_=14.3 *** |
|  | interaction | F_1,36_=5.58 * | n.s. |
|  |  |  |  |
| Shoot P content | light | F_1,36_=234 *** | n.s. |
|  | inoculation | n.s. | F_1,36_=30.4 *** |
|  | interaction | F_1,36_=8.76 ** | F_1,36_ =15.8 *** |
|  |  |  |  |
| Root P content | light | F_1,35_=251 *** | F_1,34_=11.6 ** |
|  | inoculation | n.s. | F_1,34_=97.3 *** |
|  | interaction | F_1,35_=9.88 ** | n.s. |
|  |  |  |  |
| Shoot P concentration | light | n.s. | F_1,36_=9.97 ** |
|  | inoculation | F_1,36_=13.32 *** | n.s. |
|  | interaction | F_1,36_= 41.1*** | F_1,36_=30.86 *** |
|  |  |  |  |
| Root P concentration | light | F_1,35_=15.1 *** | n.s. |
|  | inoculation | F_1,35_=21.5 *** | F_1,34_=165 *** |
|  | interaction | F_1,35_=19.1 *** | n.s. |
|  |  |  |  |
| Shoot N content | light | F_1,16_=773 *** |  |
|  | inoculation | n.s. |  |
|  | interaction | n.s. |  |
|  |  |  |  |
| Root N content | light | F_1,16_=1706 *** |  |
|  | inoculation | n.s. |  |
|  | interaction | F_1,16_=35.4 *** |  |
|  |  |  |  |
| Shoot N concentration | light | F_1,16_=422 *** |  |
|  | inoculation | n.s. |  |
|  | interaction | F_1,16_=32.1 *** |  |
|  |  |  |  |
| Root N concentration | light | F_1,16_=45.9 *** |  |
|  | inoculation | n.s. |  |
|  | interaction | n.s. |  |
|  |  |  |  |
| Shoot δ^15^N | light | F_1,16_=48.4*** |  |
|  | inoculation | n.s. |  |
|  | interaction | F_1,16_=51.2 *** |  |
|  |  |  |  |
| Root δ^15^N | light | F_1,16_=166 *** |  |
|  | inoculation | F_1,16_=20.3 *** |  |
|  | interaction | n.s. |  |
|  |  |  |  |
| Root-to-shoot biomass ratio | light | F_1,36_=184 *** | F_1,36_=16.8 *** |
|  | inoculation | F_1,36_=12.7 ** | n.s. |
|  | interaction | F_1,36_=8.43 ** | n.s. |
|  |  |  |  |
| Number of branches | light | F_1,36_=177 *** | n.s. |
|  | inoculation | n.s. | F_1,36_=7.74 ** |
|  | interaction | n.s. | n.s. |
|  |  |  |  |
| Length of axis | light | F_1,36_=66.6 *** | F_1,36_=26.5 *** |
|  | inoculation | n.s. | F_1,36_=16.4 *** |
|  | interaction | n.s. | n.s. |
|  |  |  |  |
| Leaflet surface | light | n.s. | F_1,36_=12.7 ** |
|  | inoculation | n.s. | F_1,36_=4.29 * |
|  | interaction | n.s. | n.s. |
|  |  |  |  |
| Shoot C concentration | light | F_1,16_=44.7 *** |  |
|  | inoculation | F_1,16_=18.7 *** |  |
|  | interaction | F_1,16_=15.1 ** |  |
|  |  |  |  |
| Root C concentration | light | n.s. |  |
|  | inoculation | n.s. |  |
|  | interaction | n.s. |  |
|  |  |  |  |
| Shoot excess ^13^C | light | F_1,16_=510 *** |  |
|  | inoculation | n.s. |  |
|  | interaction | n.s. |  |
|  |  |  |  |
| Root excess ^13^C | light | F_1,16_=597 *** |  |
|  | inoculation | n.s. |  |
|  | interaction | F_1,16_=5.76 * |  |
|  |  |  |  |
| Root-to-shoot excess ^13^C | light | F_1,16_=338 *** |  |
|  | inoculation | n.s. |  |
|  | interaction | F_1,16_=5.75 * |  |
|  |  |  |  |
| Substrate excess ^13^C | light | n.s. |  |
|  | inoculation | n.s. |  |
|  | interaction | n.s. |  |
|  |  |  |  |
| *Rhizophagus* – hyphae | light | F_1,18_=25.1 *** | n.s. |
|  |  |  |  |
| *Rhizophagus* – arbuscules | light | n.s. | n.s. |
| *Rhizophagus* – vesicles | light | F_1,18_=29.2 *** | n.s. |
|  |  |  |  |
| Mycorrhizal shoot biomass response | light | F_1,18_=7.17 * | n.s. |
| Mycorrhizal root biomass response | light | F_1,18_=16.1 *** | n.s. |
| Mycorrhizal shoot P content response | light | F_1,18_=37.1 *** | F_1,18_=35.7 *** |
|  |  |  |  |
| Mycorrhizal root P content response | light | F_1,18_=16.5 *** | n.s. |
|  |  |  |  |
| Mycorrhizal shoot P concentration response | light | F_1,18_=117*** | F_1,18_=57.4 *** |
|  |  |  |  |
| Mycorrhizal root P concentration response | light | F_1,18_=19.0 ***. | n.s. |

## Supplementary Figures


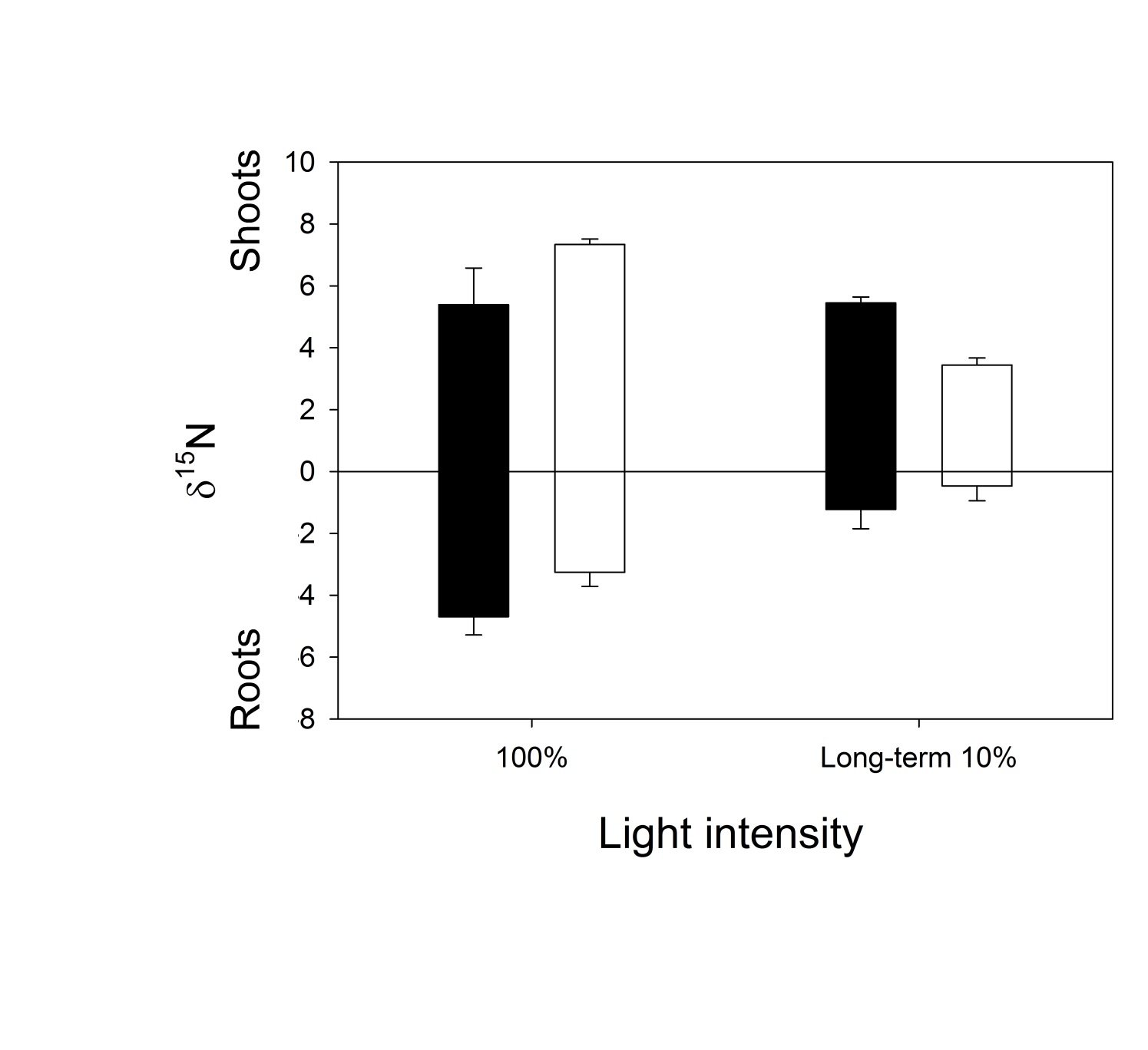


**Supplementary Figure 1. ^15^N-isotopic signature of the nitrogen in shoots and roots of the *Medicago truncatula* plants inoculated or not with *Rhizophagus irregularis* (black and white bars, respectively), and subjected or not to long-term shading.** Mean values ± s.d. (n=5) are shown.

**
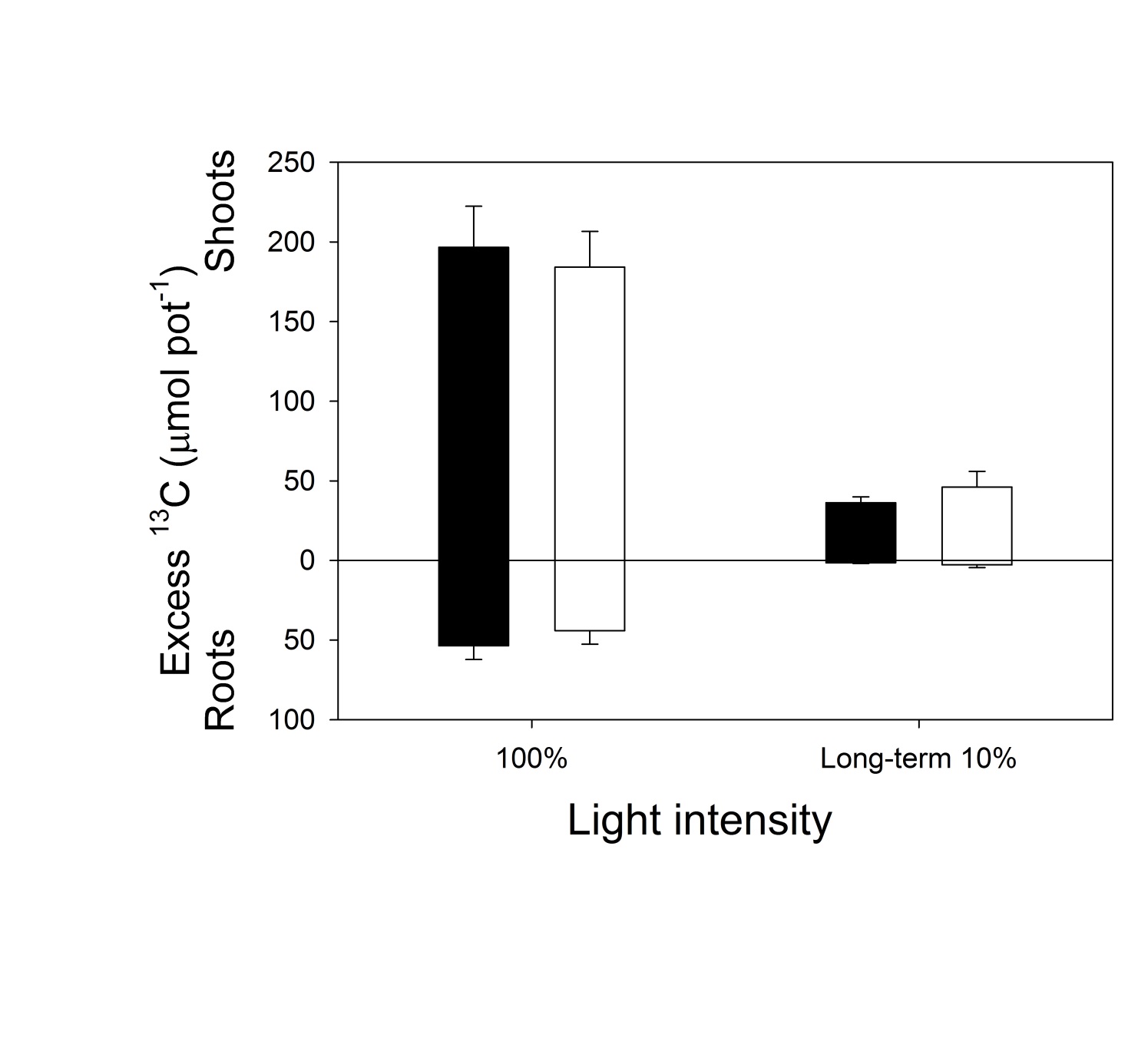
**

**Supplementary Figure 2. Amount of ^13^C originating from the heavy carbon pulse (i.e., excess ^13^C) in shoots and roots of *Medicago truncatula* plants inoculated or not with *Rhizophagus irregularis* (black and white bars, respectively), and subjected or not to long-term shading.** Mean values + s.d. (n=5) are shown.


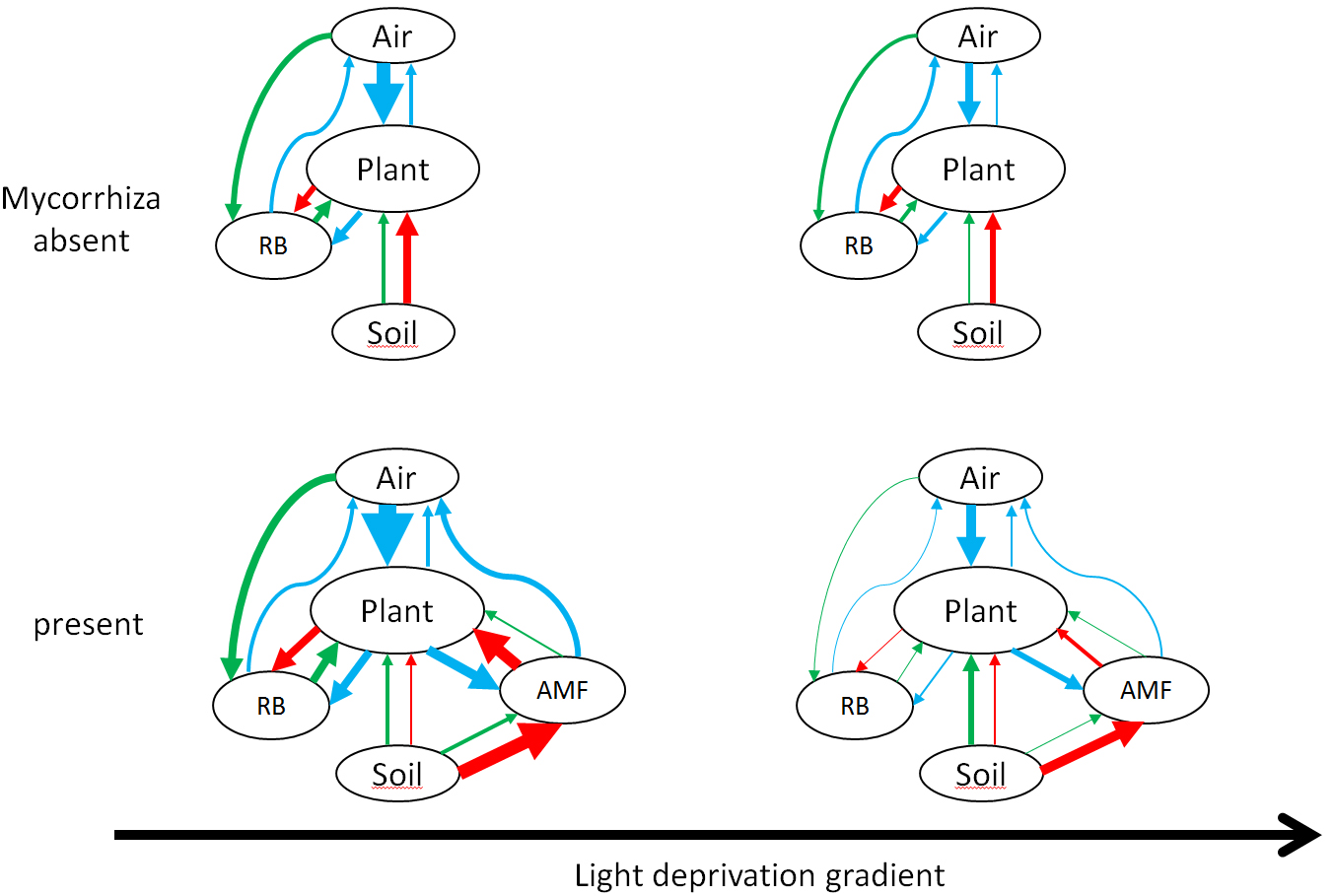


**Supplementary Figure 3. Conceptual model of major carbon (blue), nitrogen (green) and phosphorus (red) fluxes in *Medicago truncatula* plants with different combination of symbionts (RB – rhizobia, AMF – arbuscular mycorrhizal fungi) and subjected to different light conditions.** Thickness of arrows represents proportions between the different channels, though the absolute values are not up to scale.
